# Antioxidants in Hops: Bioavailability, Health Effects and Perspectives for New Products

**DOI:** 10.3390/antiox11020241

**Published:** 2022-01-27

**Authors:** Corina-Aurelia Zugravu, Roxana-Elena Bohiltea, Teodor Salmen, Elena Pogurschi, Marina Ruxandra Otelea

**Affiliations:** 1 Department of Hygiene and Ecology, “Carol Davila” University of Medicine and Pharmacy, 050463 Bucharest, Romania; corina.zugravu@umfcd.ro or; 2 Department of Obstetrics and Gynecology, “Carol Davila” University of Medicine and Pharmacy Bucharest, 020021 Bucharest, Romania; roxana.bohiltea@umfcd.ro or; 3 Department of Diabetes, Nutrition and Metabolic Diseases, “Prof. Dr. N.C.Paulescu” National Institute of Diabetes, 030167 Bucharest, Romania; 4 Faculty of Animal Productions Engineering and Management, University of Agronomic Sciences and Veterinary Medicine of Bucharest, 57 Marasti Blvd, 011464 Bucharest, Romania; elena.pogurschi@usamv.ro or; 5 Clinical Department 5, “Carol Davila” University of Medicine and Pharmacy, 050463 Bucharest, Romania; marina.otelea@umfcd.ro or

**Keywords:** hops, antioxidants, metabolic syndrome, bioavailability

## Abstract

Hop plant (*Humulus lupulus* L.) has been used by humans for ages, presumably first as a herbal remedy, then in the manufacturing of different products, from which beer is the most largely consumed. Female hops cones have different useful chemical compounds, an important class being antioxidants, mainly polyphenols. This narrative review describes the main antioxidants in hops, their bioavailability and biological effects, and the results obtained by now in the primary and secondary prevention of several non-communicable diseases, such as the metabolic syndrome related diseases and oncology. This article presents in vitro and in vivo data in order to better understand what was accomplished in terms of knowledge and practice, and what needs to be clarified by additional studies, mainly regarding xantohumol and its derivates, as well as regarding the bitter acids of hops. The multiple protective effects found by different studies are hindered up to now by the low bioavailability of some of the main antioxidants in hops. However, there are new promising products with important health effects and perspectives of use as food supplements, in a market where consumers increasingly search for products originating directly from plants.

## 1. Introduction

Hops Humulus Lupulus Linnaeus is a plant known and cultivated by humans for a very long time. Early evidence links its origin to ancient China, later found in most temperate areas of the world [1]. Though today we associate hops with beer production, and this was true for centuries, its initial use has been different. Hops were often used as medicinal plants in the popular pharmacopoeia, designed to relieve the symptoms of a large number of health problems [2]. It has been attributed anti-inflammatory and antimicrobial properties, as well as diuretic, digestive, sedative, progestogenic properties, even being considered a cure for insomnia. For this wide range of health benefits, it was regarded as a life prolonging plant [3].

**Figure 1:**
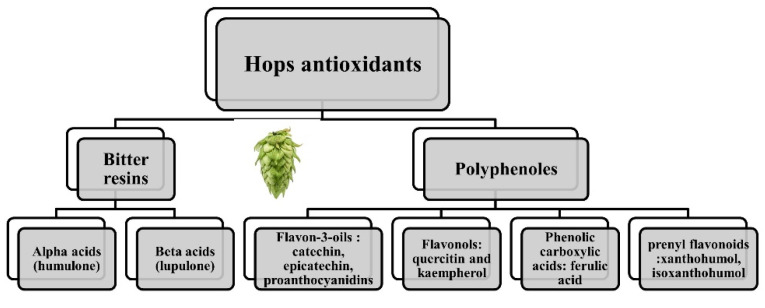
Antioxidants in hops.

The main component of the hops used today is the cone, the female inflorescence of the plant, but other areas of the hops plant also prove to be useful (the leaves, stems or rhizome). There are several bioactive molecules that underlie the sanogenetic effects of the plant [1,4,5]. For example, in the cone glands, the primary metabolites are the bitter resins and the aromatics. The secondary substances belong to three categories of substances: resins, oils and polyphenols (4–14% dried weight) [6,7] (Figure 1).

There are two types of bitter resins: alpha acids (mainly humulone) and beta acids (mainly lupulone). As for polyphenols, they can be classified into: flavonols (quercitin and kaempherol), flavon-3-oils (the main ones being catechin, epicatechin, proanthocyanidins), phenolic carboxylic acids (ferulic acid) and, in lesser quantities, prenyl flavonoids such as xanthohumol (0.1–1% on dry weight) [8,9] and isoxanthohumol. Their very small quantities do not necessary preclude a biological effect. For example, isoxanthohumol is transformed in the intestine into prenylnaringenin, a phytoestrogen uptaken by the intestinal cell. Depending on the individual microbiota, it was estimated that one third of the population could produce high enough levels to reach the threshold for a biological effect [10].

The bioactive components of hops are still under research, due to their antioxidant, anti-inflammatory and immunomodulatory effects. Their primary or secondary prevention properties regarding some chronic, degenerative diseases with great prevalence in the contemporary world gave them a special importance. Most studies focus on the action of prenylflavonoids (xanthohumol and its derivatives), which were considered, until recently, the only substances of pharmaceutical importance in hops [11].

## 2. Bioavailability of the Active Substances

As for all foods, bioavailability is crucial when evaluating the practical consequences of hops components. Studies show that the bioavailability is rather low [12,13], and ways for increasing it were developed. One alternative is prenylation, which increases the biological activity of dietary flavonoids by enhancing their affinity for estrogen receptors, facilitate the interaction with cellular membranes and proteins. As a consequence, they tend to remain for longer time in certain target cells and have greater antioxidant effect [14]. The bioavailability is increased in the presence of the prenyl group, which decreases the polarity of flavonoids. Degradation of xanthohumol in the intestine is also influenced by the prenyl group. The presence of this group slows down the degradation of xanthohumol [14,15].

**Figure 2:**
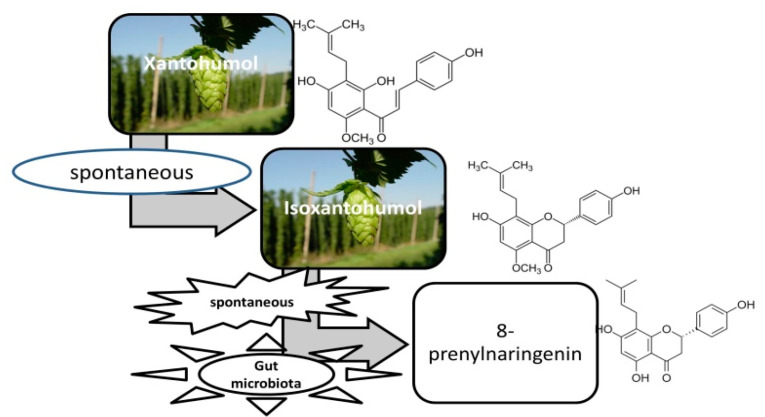
Transformation of xantohumol in the digestive system.

The bioavailability of prenylflavonoids is not only influenced by the presence or absence of functional groups. The human microbiota, as stated above, greatly influences the resulting metabolites (Figure 2), which may be of pharmaceutical importance as well. Numerous studies have been performed to identify them, and less to assess their bioavailability.

Depending on the predominant microorganisms in microbiota, the metabolic products may be different, with an activity similar or different to that of the initial substances [16].

Xanthohumol, the main substance studied among the many components of hops, has a low bioavailability to oral administration, which limits its sanogenetic effects [13]. Nookandeh et al. showed that more than 80% of the products of the intestinal degradation of the orally administered xanthohumol to laboratory animals was excreted in feces [17]. Similar results were found by Avula et al. who concluded that most of the administered xanthohumol is excreted in feces in the 24 hours after administration. Studies conducted on Caco-2 cells showed that xanthohumol accumulates in the cytosol, binds to the cellular proteins [18] and even has the ability to insert inside the lipid bilayer of cell membranes, which makes a rapid transmembrane transfer very difficult [19,20,21]. 

Novak et al. investigated the pharmacokinetics and bioavailability of pure xantohumiol or a hops extract, comparing the oral with the iv routes in rats. There were no differences due to sex, but the oral absorption was very low compared with the iv route. The probability of a hepato-entero-hepatic recirculation of the compounds was also postulated, based on the peaks of plasma levels after ingestion [22].

A blinded randomized study with different doses of xanthohumol given orally showed similarities with the animal experiments. This study highlighted that plasma xanthohumol significantly increases only after more than 60 mg. Two peaks in plasma xanthohumol (as observed in animal experiments), 1 h and 4 h after administration, were observed; the half time was around 20 h [23].

Low bioavailability was noted also for Iso-α-acids (IAA) and the reduced derivates, dihydro-iso-α-acids (DHIAA) and tetrahydro-iso-α-acids (THIAA). However, reduced metabolites had a higher bioavailability [24].

Cattoor et al. also carried out an analysis of the absorption of bitter acids from hops through Caco-2 monolayers. Membrane permeability was higher for alpha than for beta acids. Consequently, oral administration of beta acids had a low absorption, probably limited by the active transporters of P-gp and MRP-2 efflux and by phase II metabolism [25].

As low bioavailability is common to many plant bioactive substances, new forms of administration and new additives are being tested to improve or address this issue in the near future.

## 3. Biological Effects

The bioactive components of hops are still in the researchers’ objective, for their role in primary or secondary prevention of some chronic, degenerative diseases with great prevalence in the contemporary world, in which oxidative mechanisms play an important role. An antioxidant is a substance that delays or prevents the oxidation of a substrate, thus reducing the oxidative stress. As reactive oxygen species (ROS) are produced during the normal metabolism, a good balance between oxidative and anti-oxidant mechanisms is necessary. Generally, the antioxidants are divided in enzymatic (e.g., superoxide dismutase, glutathione reductase, catalase, peroxidase, etc.) and nonenzymatic (e.g., glutathione, vitamins).

The oxidative stress is enhanced in many pathological circumstances. Activation of the inflammatory cells during defense against microorganisms [26], cells with low supply in oxygen in chronic or acute ischemia, excess NO formation as in autoimmune diseases [27] or allergies [28], are circumstances of excess oxidative compounds production.

The type of hops, the extraction method of the antioxidant substances and the assays used to assess the antioxidant power (the radical scavenging ability or the ability to inhibit lipid oxidation under accelerated conditions) are some of determinants of the variability in the results when evaluating hops antioxidative efficacy [29,30,31,32].

However, these data should be interpreted with caution, and cannot be fully extrapolated to real life, as many of the substances in hops act synergistically and combine their antioxidant effects, with the anti-inflammatory or antiproliferative ones.

Most studies focused on the action of prenylflavonoids (xanthohumol and its derivatives) which were considered, until recently, the only substances of pharmaceutical importance [11]. More recently, studies on the action of bitter acids (humulone and lupulone) utilized as brewing additives have also given interesting results [11].

In this review, we will focus on two pathological conditions associated with oxidative stress in which a possible role of the hops compounds have been investigated: cancer and metabolic syndrome.

## 4. Hops Antioxidants and Cancer

Hops substances and cancer have been extensively researched during time and from the multitude of components, xanthohumol was frequently under scrutiny.

The substance was discovered in 1957 [33], its properties being brought to light only in the recent decades [34]. After ingestion, xanthohumol undergoes transformations, sometimes resulting in substances with a higher antioxidant power and a wider spectrum of action. It was noticed that xanthohumol is transformed by a non-enzymatic mechanism in isoxanthohumol and enzymatically, in 8 and 6-prenylnaringenin and in desmethylxanthohumol [35,36].

The positive action of xanthohumol in neoplasms was shown for tumors with various localizations, from lung or digestive system (colon, pancreas), to endocrine (thyroid) or genito-urinary (cervical, ovarian), head and neck, skin, malignant melanoma or leukemia [37,38].

The ways in which xanthohumol acts were the subject of a large number of studies are summarized by Jiang et al. in a 2018 article [37].

The action of xanthohumol is probably achieved by the inhibitory action on two signaling pathways with an essential role in maintaining the appearance of malignancy and in achieving metastases: Akt and NF-κB. Xanthohumol induces apoptosis of cancer cells by stimulating pro-apoptotic proteins (Bax, PARP, AIF- caspase-3, -8, -9) and by inhibiting proliferation, achieved by inhibiting Notch1, mTOR, STAT3. Xanthohumol also acts on the migration and invasion of neoplastic cells, probably by inhibiting FAK and MMP-2 expression [37]. Moreover, xanthohumol seems to act synergistically with the usual chemotherapeutic treatments, which is certainly a positive factor because it could theoretically allow for a decrease in the doses administered. Jiang et al. note that xanthohumol influences various proteins that bear upon proliferation, migration, invasion, apoptosis or resistance to many chemotherapeutics in cancer patients, without the exact mechanisms being known at this time.

Girisa et al. conclude that hops have an important anticancer potential, acting by modifying both signaling pathways (Akt, AMPK, ERK, IGFBP2, NF-κB, and STAT3), and by modulating proteins (Notch1, caspases, MMPs, Bcl-2, cyclin D1), oxidative stress markers, miRNAs and tumor suppressor proteins [39].

**Figure 3:**
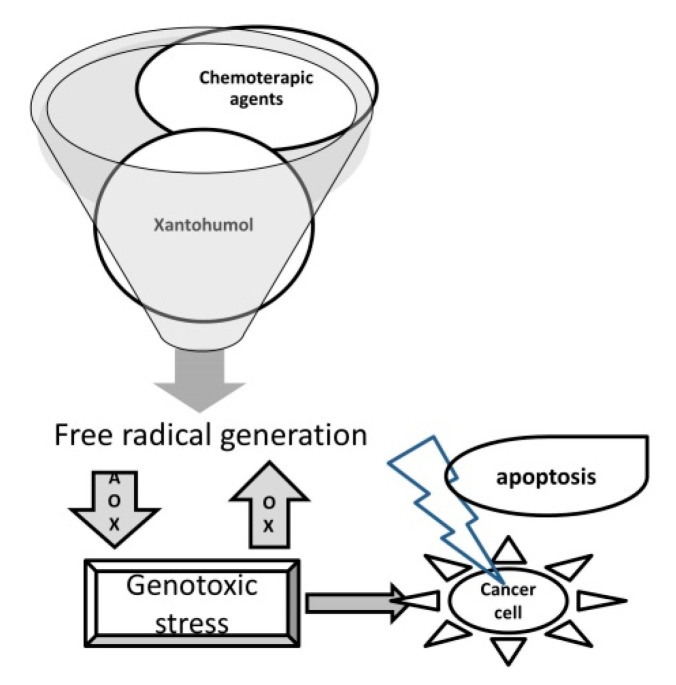
The oxidative stress: a common mechanism of action of xantohumol and chemotherapeutics on cancer cells.

A very interesting aspect is related to the antioxidant action of xanthohumol. It is known that in the etiology of neoplasms, from cell proliferation to local invasion and metastases, oxidative stress is involved, on the regulation of which many oncological treatments are based. It is just that the accumulation of free radicals is not only a cause, but also a factor that determines the sensitivity to treatment [40], many chemotherapeutic agents acting precisely to stimulate the formation of free radicals in neoplastic cells [41]. In this context, it was shown that, although it has a high antioxidant power, xanthohumol also induces an important production of free radicals in various neoplastic cell lines, causing their apoptosis [42,43,44] (Figure 3).

Wei et al. concluded that xanthohumol also causes an imbalance between intracellular antioxidants, such as SOD, and the production of free radicals, which potentiates xanthohumol’s apoptotic and antimetastatic, antiproliferative action. High levels of free radicals, which are easily reached by depressing cellular antioxidant systems, reduce NF-κB activity, leading to cell death [45]. 

Xanthohumol stimulated the anticancer actions of high ROS by the NF-κB signaling pathway. Future studies should show the extent to which classical neoplastic treatments could benefit from the associated administration of xanthohumol products in clinical practice.

An important activity of xanthohumol is related to metastasis formation. After xanthohumol proved to be effective in pulmonary adenocarcinoma, Sławińska-Brych et al. also confirmed its effectiveness in inhibiting metastatic processes. The mechanisms involved in the antiinvasive action appear to be the inhibition of the ERK/MAPK pathway and the suppression of FAK and PI3/AKT signaling [46].

Other mechanisms seem to be common to xanthohumol, iso-xanthohumol and 8-prenylnaringenin, as antioxidant and anticancer agents. They refer to the action on Aldose reductase (AKR1B1) and a counterpart, AKR1B10. Both substances are overexpressed in neoplasms with various localizations, from lung and prostate, to breast. This makes them potential treatment targets. Seliger et al. showed that xanthohumol, iso-xanthohumol and 8-prenylnaringenin substantially and indiscriminately inhibit B1 and B10 [47].

Recently, new studies reveal the action of xanthohumol in neoplasms with localizations that had not been previously followed. One of these is the stomach. Gastric cancer is a form of cancer with a high incidence and a very poor prognosis. Wei et al.’s 2018 study confirms that xanthohumol has a deleterious effect on gastric cancer cells, but not on healthy gastric cells. Xanthohumol inhibits proliferation, induces apoptosis and reduces cancer cell metastases, most likely by free radical overproduction [44].

An interesting subject could be the effects of xanthohumol in colorectal cancer, from the perspective of the previously mentioned reduced bioavailability, which leaves most of the ingested substance in contact with the terminal areas of the digestive system. Scagliarini et al. show that xanthohumol, applied on cancer cell cultures, can activate cell cycle disruptions, even in non-toxic concentrations, by reducing cyclins A and B and concomitantly increasing cyclin E [48]. Apoptosis was found in some of the most resistant cell lines 48 h after the debut of treatment with xanthohimol, and their sensitization to the action of usual chemotherapeutics was also noticed. Xanthumol activated p53 and p21, which recommends the substance as a pre-treatment of chemotherapy, not only to increase its effectiveness, but also to reduce the therapeutical dose and, consequently, the side effects. Taking into account the conclusions of Ambroz’s study [49], which reports antagonistic effects on concomitant administration of xanthohumol/chemotherapy, it was concluded that administration should be staged, in order to avoid decreasing the efficacy of the chemotherapeutics. It should be noted that other studies confirmed the cytotoxic action of other prenylflavonoids in hops on colonic proliferative cells, but xanthohumol had the most active contribution [50].

Harish et al. [12] summarized the mechanisms in which xanthohumol exerts its antineoplastic action in vitro, as follows: induction or inhibition of apoptosis, modulator of oncogenic signaling pathways, the free radical formation, and the ERK1/2, NF-κB, and Akt signals. The major courses of action differ after the location of the cancer. For example, in breast cancer it acts by decreasing the expression of Notch1, survivin and Ki-67, and by potentiating the expression of capsase 3, simultaneously inhibiting STAT3 and MDR 1. Another neoplastic localization in which xanthohumol was effective is the prostate, where it induced apoptosis via TNF-Related Apoptosis Inducing Ligand as well as by depolarizing mitochondria by activating procaspases 3, 8 and 9. One has to take into consideration difficulty of in vivo assessments, especially due to the reduced bioavailability of oral administration.

Recently, minor components of the antioxidant profile of hops were highlighted and investigated, having synergistic effects and being sometimes more active than xanthohumol. One of the minor natural compounds is xanthohumol C, which inhibits mammary neoplastic cell lines more intensely than xanthohumol or xanthumol-enriched hops extract. The mechanism of action of xanthohumol C appears to be different from that of xanthohumol; it seems to be involved in endoplasmic reticulum stress and in altering the adhesion of cells to each other [51]. Xanthohumol C is a chalcone with various types of action, including cytotoxic [52] and antioxidant [7,53] manifested in the context of neoplasms. Xanthohumol C was described by Stevens et al. [54] and due to difficulties in obtaining pure substances and, consequently, to the interference of other minor compounds, they noticed a lower antiproliferative action in certain neoplastic cell lines (breast, colon, prostate) than the one of xanthohumol [52].

Other hops substances were studied in relation to neoplasms, especially the bitter acids. Humulon shows anticancer and anti-inflammatory effects on skin cancer [55]. Lupulon activates apoptotic pathways including apoptotic TRAIL-receptors, in human cancer colon cells and in the equivalent metastatic cells, even in TRAIL resistant cancer cells [56,57]. Both substances show promising effects for cancer prophylaxis and treatment. 

## 5. Hops and the Metabolic Syndrome

Metabolic syndrome is a cluster of risk factors for diabetes mellitus and cardiovascular diseases in the presence of central obesity. The criteria for diagnosis metabolic syndrome are the expression of the impairment of lipid and glucose metabolism, insulin resistance and the endothelial dysfunction. All these pathological findings have in common excessive ROS formation. In the following lines, we will present data on how the compounds of hops could influence the components of the metabolic syndrome. 

### 5.1. Obesity

As mentioned above, central obesity (clinically assessed by the abdominal circumference) is a key element of the metabolic syndrome. Obesity induces systemic oxidative stress through superoxide generation, enhanced oxidative phosphorylation, reduced activity of anti-oxidant enzymes, hyperleptinemia, and activation of the polyol and hexosamine pathways [58]. In this respect, reduction in body adipose tissue would contribute to the homeostasis of the oxidative status.

In animal experiments, xanthohumol was able to reduce body weight, lipid absorption and plasma glucose [59,60]. Metabolomic studies showed that xanthohumol induces a catabolic status [61]. In this experiment, the effect of xanthohumol was dose dependent; while in low doses the uncoupled effect was the main consequence, at higher levels xanthohumol inhibited the mitochondrial respiration in white and brown preadipocytes and in the muscle cells. The energy depletion activates energy production mechanism other than the mitochondrial ATP, such as the creatine kinase system, lactate production from pyruvate, glycolysis and the degradation of the macronutrient complexes, finally leading to the anti-obesity effects. 

Another interesting study showed that xanthohumol and tetrahydroxanthohumol suppress diet-induced accumulation of lipid in liver, in the subcutaneous and in the mesenteric adipose tissue in mice [62]. This effect was partially due to an increase in energy expenditure. The study also revealed the affinity of xanthohumol and tetrahydroxanthohumol to peroxisome proliferator-activated receptor γ (PPARγ), with an inhibitory effect of the metabolic pathways induced by this transcription factor. Another positive effect on body weight of the PPARγ inhibition is the suppression of the adipocyte’s differentiation. Referring to the oxidative status, PPARγ is generally considered a modulator with preponderant antioxidative role; PPARγ initiates the transcription of the catalase, manganese superoxide-dismutase and inhibits of the pro-oxidative inducible NO-synthetase and cyclooxygenase-2 [63]. Whether the antioxidative effect of body mass reduction induced by hops compounds will prevail or not on their inhibitory effect of PPARγ remains to be established by future research.

Xanthohumol prevents lipid oxidation in liver, as shown in an experiment with carbon tetrachloride in rats [64]. The non-alcoholic fatty liver disease, a clinical entity linked to the metabolic syndrome, also involves the lipid oxidation among other pathogenic mechanisms and the inhibition of this process induced by xanthohumol might be of benefit. In fact, the xanthohumol has been investigated for the possible prevention of lipid and bile acids accumulation in liver [65]. It has been shown that xanthohumol ameliorates HFD-induced hepatic injury and the dysfunctional lipid and bile acid metabolism. 

### 5.2. High Density Lipoprotein Cholesterol (HDL-c)

HDL-c reduction is an indirect marker of the reverse transport of cholesterol for hepatic processing and biliary excretion. Though, if HDL-c is low, cholesterol accumulates in peripheral tissues (e.g., vascular, pancreas, muscle) contributing to atherogenesis, impaired insulin secretion and insulin resistance. Besides the cholesterol transport, HDL-c also has a significant antioxidative activity on low density lipoprotein cholesterol (LDL-c) and also on the cellular membranes of erythrocytes and astrocytes [66]. The metabolic syndrome implies not only a diminished quantity of HDL-c, but also a reduction in the antioxidant capacity of this lipoprotein [67]. HDL-c directly protects LDL-c from oxidation induced by ROS and by removing oxidized lipids from LDL-c; therefore, the amount of oxidized LDL-c, the major initiator of the inflammatory process in atherosclerosis, is reduced [66].

Isohumulone is able to raise the HDL-c in plasma and reduces the atherogenic index in a dose–dependent manner, at least in experimental models [68]. The mechanism seems to be mediated by the activation of the PPARα, which has a significant role in increasing the transfer of cholesterol from VLDL/IDL to the HDL-c through increasing the hepatic expression of the phospholipid transfer protein [69].

Even if the antioxidant activity of HDL-c on the oxidized LDL-c has not been directly assessed, the inhibition of this oxidative process was demonstrated, as prenyl flavonoids extracted from hops were able to suppress, in vitro, the oxidation of low-density lipoproteins induced by peroxynitrite [70].

### 5.3. Triglycerides (TG)

TG metabolism is impaired in metabolic syndrome. The visceral adiposity increases the release of free fatty acids in the portal system and the production of TG in hepatocytes. In insulin resistance status, the hepatic TG synthesis is promoted and the high plasma levels decreases the clearance of the VLDL [71]. The TG-rich lipoproteins release in the peripheral endothelium neutral and oxidized free fatty acids that propagate the local inflammation and damage of the vascular wall [72]. In experimental studies, xanthohumol lowered the plasma TG even in rats fed with a high fat diet [60]. The mechanisms revealed by this study were the reduction in hepatic fatty acid synthesis and the enhanced excretion of TG through feces, probably by a decline in lipid absorption. They also confirmed previous experimental data about the reduction of the SREBP1c expression in hepatic cells [73], a nuclear receptor which controls the fatty acid and TG synthesis in the liver [74]. Similar effects were obtained with another isomerized hops extract, in which the most active compound was isohumulones [75]. The mechanism was related to the diminution in lipid absorption, secondary to the inhibition of the pancreatic lipase [76]. The effect of beer and/or the beer compounds on the pancreatic exocrine secretion is still debatable and very much depends on the methods used to estimate in vitro the level and the content of the secretion. Some authors suggested that the stimulatory effect of the non-alcoholic components of beer (e.g., for the pancreatic amylase production) are neutralized by the direct inhibitory effect of circulating ethanol [77].

Activation of PPARα, as previously mentioned, lowers the hepatic TG stores and the suppression of TG secretion from hepatocytes [78], but these effects depend on the genetic polymorphisms of the PPARα gene and on the epigenetic modifications related to the interaction with the environmental factors [79]. To the best of our knowledge, the epigenetic modifications induced by hops compounds on the PPARα gene expression have not been studied yet.

Taken together, these data assemble in a positive effect on the lipid metabolism related to the different compounds of hops. The translation of these experiments to preventive measures needs definitely more research. In a prospective cohort of more than 8000 persons, beer consumption was associated with metabolic risk, but there was a large variation according to the level of consumption. In the multivariate analysis, the OR for moderate consumption (<1–2 drinks/week was 1.07 (CI = 0.75–1.52) compared to the non-drinkers, but for heavy drinkers (≥7 drinks/week) the OR increased significantly 1.56 (CI = 0.94–2.62). In what concerns the lipid profile, heavy drinkers had higher TG, but lower risk for low HDL-c in plasma. Unfortunately, the authors did not report if there is any point in which there is no negative influence on TG and the positive effect on HDL-c is still present.

### 5.4. Glycaemia

Impaired fasting glycemia or impaired glucose tolerance define prediabetes, a condition frequently associated with the metabolic syndrome. In a randomized trial, a 4-week treatment with isohumolone in prediabetic patients significantly reduced the fasting glucose and the HbA1C as compared to placebo [80].

Intermittent high glucose environment stimulates superoxide production and enhances endothelial cell apoptosis to an even greater extent than exposure to a constant high glucose level [81,82].

Hops compounds reduce the oxidative status related to hyperglycemia in a direct or indirect mode. For example, xanthohumol seems to have a direct effect on the systemic and local (endothelial, renal, liver) oxidative status, as noticed with beer supplemented with xanthohumol administered to diabetic rats. In this experiment, xanthohumol increased the activity of glutathione reductase, which, in turn, increases the ratio of reduced/oxidized glutathione. Xanthohumol also normalized the level of H_2_O_2_, reduced the 3-Nitrotyrosine and the carbonylation of proteins [83], showing an interference with many antioxidative mechanisms. Indirect effects refer to the capacity of different hops compounds to reduce glycemia. For example, in mice fed on a high fat diet, tetrahydroxanthohumol significantly improved the glucose clearance, reducing the time and peak of hyperglycemia [62]. In another experiment, xanthohumol was able to reduce glycemia in a dose dependent manner, but this effect was noticed only in male rats [59]. Data about the mechanisms by which xanthohumo decreases glycemia continue to emerge. Reduction in the hepatic glucose output is a possible mechanism. Xanthohumol acts as a ligand for the farnesoid X receptor (FXR), a nuclear receptor that regulates genes involved in gluconeogenesis [73]. Another mechanism is the reduction in carbohydrates absorption. Indeed, inhibition of the amylase [76] and α-glucosidase [84] reduces the absorption and promotes the excretion of carbohydrates in feces. Regarding other components of hops, isohumulones seem to have a positive effect in correcting hyperglycemia [80].

## 6. Safety and Prospects

Scientists try to develop new products based on hops that capitalize on its proven sanogenetic effects. They take into account the studies regarding the antioxidant effects of hops’ components, correlated with the consumer’s desire to have products of natural origin. Moreover, in addition to hops fruits, the use of hops leaves with a high antioxidants content is also considered [85]. Of course, most of the new products benefit not only from the antioxidant effect, but also from other positive actions of the existing substances in hops, with convergent effects. Thus, the antioxidant effect is often combined with anti-inflammatory, antimicrobial and antifungal effects.

An important discussion refers to the possible excess of antioxidants in diets, especially related to influencing neoplasms [86,87]. Of course, the bioavailability of many antioxidants is quite low when administered through food. Today, however, there are supplements that ensure increased bioavailability and which can be used in much larger quantities than anticipated, leading to the entry of high levels of antioxidants into metabolism. Thus, their safety can be questioned. As for the ingredients of hops, they have been evaluated in relation to consumers’ safety and it was concluded that the extracts and bitter acids are safe when used short term and in adequate quantities, such as 300 mg/day for extracts, for a period of 3 months and 35 mg/day for bitter acids, for the same duration [88]. The safety of xanthohumol was studied in vitro and in vivo (on rats) in the context of its use as an antineoplastic agent. It was noticed that the substance selectively targets cancer cells, not raising major problems with short-term administration in high doses [89,90,91].

Another aspect to consider is the possible interference of hops substances in drugs metabolism. Prenylflavonoids interfere with the activity and expression of phase II enzymes in Caco2 cells, modulating the expression and activity of glutathione S-transferase and catechol-O-methyltransferase and inhibiting sulfotransferase. The expression of uridine diphosphate (UDP)-glucuronosyl transferase 1A6 mRNA was potentiated and that of glutathione S-transferase 1A1/2 was inhibited [50].

These elements must be taken into account when prescribing hops supplements with concomitant medicinal products.

As with other phytotherapeutic products, the best way is to eat foods or supplements in physiological doses [92], and from this point of view, hops is considered a useful plant when designing functional products. Overcoming problems of bioavailability is still a challenge.

When developing a hops-based product, the first element to consider is the use of hops with a maximum content of antioxidants. The quantitative and qualitative level of antioxidants varies depending on different factors. Thus, there are different varieties of hops with different levels of antioxidants [5]. The timing of harvesting hops is also an important factor [93]. Subsequently, the processing of hops, from drying to heat treatment, also changes the level of antioxidants. For example, there are studies that show that their level is higher in fresh hops, although drying does not induce major losses [94]. The implementation of some biotechnologies for the modification of hops is taken into consideration, resulting in high levels of xanthohumol and/or 8 prenilnaringenin. These types of hops can be utilized for the production of beer, but also of some supplements [8].

Finally, if antioxidants are separated from hops for use in supplements or fortified foods, the extraction methods can play an important role in the level and types of antioxidants obtained [4,5].

The utilization of hops and its components in functional foods is not, to date, supported by any health claim under relevant European legislation. There is not, at least so far, an accepted claim, and the only evaluated and rejected one refers to a supplement that used the phytoestrogen activity of prenylnaringenin [95]. 

However, there is a potential to see products with health claims and not only in the category of supplements, but also in food (e.g., beer) as studies in the field will provide new and conclusive data [96,97].

**Figure 4:**
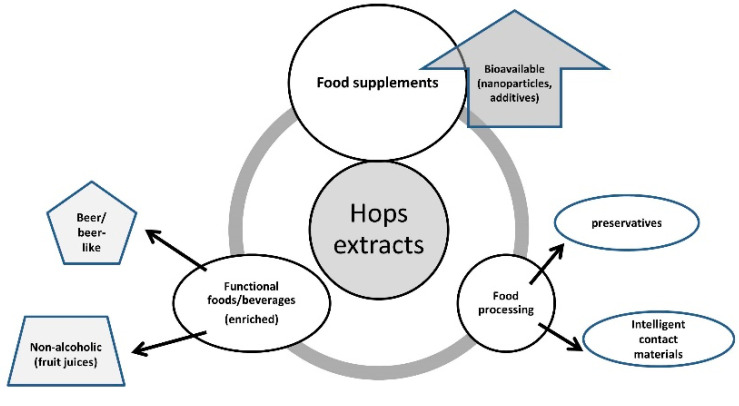
Perspectives for hops based products.

Some of the perspectives for hops-based products are presented in Figure 4.

To date, the use of infusions or extracts of hops in food has been investigated, as antioxidants that increases the stability of some essential components (proteins, fats) and some important organoleptic elements (color). In fact, in beer, hops have a preservative action due to the antioxidant capacity [98]. Villalobos-Delgado et al. [99] confirmed preservative effects, especially of hops in powdered form, when applied to raw or prepared meat products, refrigerated or frozen. During storage at low temperatures, the action of oxidases in meat continues, albeit at a slower rate. Even at this level, the blocking effect of hops is noticed. The use of the antioxidant action of hops in preserving foods, thus prolonging their shelf life, has a lot of potential, especially for meat products, where it increases not only their freshness, but also their functional properties [100,101,102].

However, taste alteration can be a barrier. In this sense, in order to avoid the bitter taste often associated with the addition of hops, new products are developed. An example is the development of lecithin-based nanoliposomes in which lupulone and xanthohumol aresimultaneosly encapsulated, with an antioxidant power that was preserved. The capsules can be used in various foods [103].

Other foods in which hops extracts were added are bakery products. Along with the antifungal action, hops extract and adjacent lactic acid bacteria were used in leavens to produce bread. The consequence was an increase in the concentration of polyphenols in bread and a rise in its antioxidant activity [104].

The antioxidants in hops were also used in the production of protective food films. Emulsion films based on different biopolymers were manufactured with the addition of alcoholic hops extract in different concentrations, resulting in films with a higher antioxidant capacity and therefore with a higher degree of protection against food spoilage [105].

Beyond such uses, hops and its components can be used in the production of functional beverages, to offer them, along with enhanced organoleptic qualities, the advantage of a significant intake of antioxidants. This trend is all the more positive, as evaluations regarding the aromatic preferences of consumers reveal the presence of hops among the first 12 favorite flavors [106].

The most promising field is ultimately the development of beer-like beverages, the staple where hops have been used for centuries.

An example of such a product is Aliophen R, an unfermented beverage based on malt and hops and possessing a polyphenol load similar to that of beers on the market. Experimental studies show that the product has an effective antioxidant and chemopreventive action, all the more important, as it can be consumed in larger quantities than beer because it does not contain alcohol [107].

Hops extract can be added to various fruit juices. In the study by Mashkour et al., hops extract was added to cherry juice, where it significantly increased the antioxidant capacity of the mixture and the sensory evaluation score [108].

New types of beer, enriched in polyphenols, from collateral products resulting in the beer industry (pellets enriched in lupulin) are also tested [109]. The result are beers that have a level of antioxidant power, up to 21.5% higher than regular beer. In these types of beer, special attention must be paid to collateral organoleptic changes, which may or may not be agreed by consumers.

The use for the enrichment of some very aromatic types of hops can fulfill both objectives: of obtaining a functional type of beer, with increased antioxidant power, and that of producing an attractive product for the consumer [110].

Beyond modified or enriched products, beer itself can be manufactured by processes that increase the level of antioxidants in the final product. Various attempts have been made in this regard [5] and the most promising seems to be a new method, based on hydrodynamic cavitation process, which allows a higher level of xanthohumol, desmethylxanthohumol and 6-prenylnaringenin to be maintained. This process was used before with other positive results, but it is also effective in obtaining beers with a higher level of antioxidants [111].

## 7. Conclusions

In conclusion, hops are a repository of bioactive substances, of promising perspectives in the prophylaxis and treatment of chronic diseases of great prevalence in the modern world. So far investigated only in vitro and in vivo on experimental animals, these substances are awaiting confirmation in clinical trials.

Along with the development of new functional products, which will provide concentrated and precise quantities of hops’ ingredients, the industry is expected to develop forms with increased bioavailability, which will solve the current problems raised by the poor absorption following the oral administration of some important antioxidants from hops.
